# Gut–X Axis and Its Role in Poultry Bone Health: A Review

**DOI:** 10.3390/microorganisms13040757

**Published:** 2025-03-27

**Authors:** Ya-Nan Lu, Tao-Jing Yue, Wen-Li Ding, Bo-Wen Xu, Ao-Yun Li, Shu-Cheng Huang

**Affiliations:** College of Veterinary Medicine, Henan Agricultural University, Zhengzhou 450046, China; 13526541233@163.com (Y.-N.L.); 15735986314@163.com (T.-J.Y.); dingwenli3011@163.com (W.-L.D.); xubowen2600@163.com (B.-W.X.)

**Keywords:** broiler chicken, bone health, leg diseases, gut microbiota, gut–bone axis

## Abstract

The normal development and growth of bones are critical for poultry health. With the rapid increase in poultry growth rates achieved over the last few decades, juvenile meat-type poultry exhibit a high incidence of leg weakness and lameness. These issues are significant contributors to poor animal welfare and substantial economic losses. Understanding the potential etiology of bone problems in poultry will aid in developing treatments for bone diseases. The gut microbiota represents the largest micro-ecosystem in animals and is closely related to many metabolic disorders, including bone disease. It achieves this by secreting secondary metabolites and coordinating with various tissues and organs through the circulatory system, which leads to the concept of the gut–X axis. Given its importance, modulating gut microbiota to influence the gut–X axis presents new opportunities for understanding and developing innovative therapeutic approaches for poultry bone diseases. In light of the extensive literature on this topic, this review focuses on the effects of gut microbiota on bone density and strength in poultry, both directly and indirectly, through the regulation of the gut–X axis. Our aim is to provide scientific insights into the bone health problems faced by poultry.

## 1. Introduction

The health of poultry bones is crucial for ensuring the overall well-being of birds. However, genetic factors, metabolic disorders, and shorter growth cycles often cause poultry to grow too rapidly. This accelerated growth leads to muscle development at a rate that can outpace bone growth, resulting in bones that may not adequately support body weight. Such conditions can disrupt bone health homeostasis [1,2]. Common bone development abnormalities in modern chicken production include osteoporosis, femoral head necrosis (FHN), and tibial dyschondroplasia (TD) [3]. In recent years, TD has been frequently studied in juvenile meat-type broilers [4,5], with incidence rates varying significantly, from 3.5% in Bulgaria to 51.7% in Denmark [3]. The prevalence of bone development issues related to TD in broiler chickens has resulted in substantial economic losses, estimated at approximately USD 150 million in the United States [6]. Conversely, osteoporosis remains the most prevalent bone disease among cage layers [7]. These conditions lead to significant financial loss and adversely affect animal welfare within poultry farming [8,9]. Importantly, the underlying prevalence of leg diseases in broiler chickens has shown an upward trend in recent years, though an increasing number of birds exhibit subclinical symptoms without visible lameness, which breeders often overlook.

Gut microbiota (GM) is a complex and dynamic population of microorganisms that constitutes the most diverse and functionally significant microbial ecosystem in the gastrointestinal tract of animals. It is closely linked to various metabolic disorders [10,11]. Recent research has shown that GM is essential for maintaining bone quantity, quality, and overall strength in poultry [12]. The gut microbiota modulates poultry bone homeostasis by secreting secondary metabolites and coordinating with multiple systems, including the immune, neurological, endocrine, and gut barrier systems, through the vascular system of the host. This interplay is referred to as the gut–X axis [13]. Cerebral nerves and enteric neurons engage in a bidirectional regulatory relationship, wherein stimuli are conveyed from enteric neurons to central neurons. This relationship can directly regulate bone formation or influence bone homeostasis indirectly through systemic circulation [14]. Studies on injury behavior in laying hens have highlighted the circulation influence of the cecum microbiota in the gut–brain axis in regulating social stress and stress-injury behavior in chicks [15]. The microbiota also mediates the modulation of bone signaling pathways through microbial-derived metabolites and ligands. For instance, GM dysbiosis has been shown to reduce calcium absorption in the intestines, leading to decreased bone strength and mass [16]. Additionally, mycotoxins can induce liver and kidney toxicity in poultry and disrupt GM homeostasis, exemplifying the gut–organ–bone axis connection [17]. Research indicates that diets containing mycotoxins in broiler chickens can alter gut microbiota, leading to a synergistic effect of multiple mycotoxins that adversely impacts bone health [17].

However, there remains a lack of clarity regarding the pathophysiological mechanisms involved in the gut–brain–bone, gut metabolites–bone, and gut–organ–bone axes connecting GM and bone health in poultry. This paper aims to propose a novel connection between the gut–X axis and bone health, using the perspective of the poultry gut–X axis as a foundational point for exploring the specific mechanisms linking gut health and bone health.

## 2. Brain–Gut–Bone Axis: Transmitting Sensory Signals That Drive Gut Microbiota

**Figure 1:**
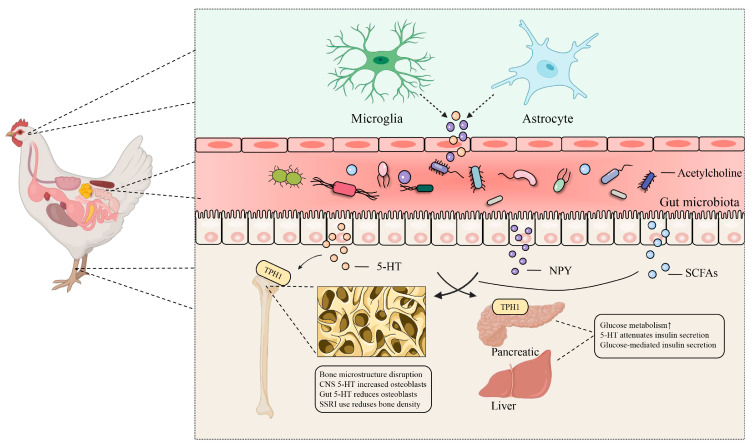
Brain–gut–bone axis: transmission of sensory signals influencing gut microbiota. Neurokines secreted by brain neurons or the central nervous system modulate gut microbiota and maintain bone homeostasis. Neurotransmitters released by glial cells and stellate keratinocytes, including 5-HT (5-hydroxytryptamine), NPY (neuropeptide Y), and acetylcholine, induce changes in intestinal microorganisms following systemic circulation. These changes subsequently impact bone homeostasis through the interactions of nerve factors, intestinal microorganisms, and their metabolites. Abbreviations: TPH1, Trypophan hydroxylase 1; SCFAs, Short-chain fatty acids.

In recent years, the role of the brain–gut axis in relation to bone health has garnered increasing attention, driven by advancements in intestinal microbiology and the in-depth exploration of gut microbiota. The brain–gut–bone axis is a complex network that facilitates bidirectional communication between the gut and the brain, involving both neurological and endocrine systems to modulate various physiological processes (Figure 1). The gut’s neuronal system is closely linked to the central nervous system through neural networks and neurotransmitters. Meanwhile, synapses in enteroendocrine cells transmit sensory signals and peripheral hormones, including neurotransmitters such as serotonin (5-HT) and dopamine, to the brain via vagal fibers that communicate gut health directly [18]. Subsequently, stress signals from peripheral and central pathways activate the hypothalamic–pituitary–adrenal (HPA) axis. The potential of this regulatory process involving the brain–gut–bone axis in enhancing bone strength is continually being explored. Probiotics have been suggested to enhance bone health by improving nutrient absorption, regulating the immune system, and modulating hormonal levels. For instance, dietary supplementation with a *Bacillus subtilis*-based probiotic in broilers resulted in greater bone mineralization and increased weights of the tibias and femurs, along with lower serum levels of C-terminal telopeptide of type I collagen (CTx) compared to control broilers after 43 days. Additionally, serotonin levels increased in the raphe nuclei, while norepinephrine and dopamine concentrations decreased in the hypothalamus, indicating that the effects of probiotics on bone health are linked to brain regulation [19]. A recent study also found that *Bacillus subtilis*-based probiotics can mitigate brain inflammatory responses in heat-stressed broilers by protecting the hippocampus through the gut–brain axis, similar to mechanisms observed in mammals [20]. Based on these studies, we can speculate that *Bacillus subtilis*-based probiotics play a protective role in bone health through the brain–gut–bone axis. It is becoming increasingly clear that the brain–gut–bone axis involves an interconnected interplay between the brain, gut, and bone systems, where signals from both the brain and gut directly or indirectly influence osteoblast activity, either individually or in combination [21]. This discussion of the brain–gut–bone axis in poultry focuses on two key aspects.

On the one hand, the brain–gut axis communicates with the gut through brain neurons and neuroendocrine factors, while the gut–brain axis relays information to the brain primarily through sensory neurons, immune mediators, intestinal metabolites, and hormones. Key mediators in this process include serotonin (5-hydroxytryptamine (5-HT)), gamma-aminobutyric acid (GABA), epinephrine, norepinephrine, dopamine, acetylcholine (ACh), neuropeptide Y (NPY), and glutamate [22]. Furthermore, the brain helps maintain bone homeostasis through a neuropeptide network regulated by the central nervous system [18]. Recent studies suggest that alterations in the gut microbiome significantly influence feather pecking and foraging behaviors in poultry [23,24,25,26]. Numerous investigations have revealed that heat stress is a major environmental stressor that compromises the intestinal and bone health of poultry [20,27,28]. Specifically, heat stress contributes to the development of osteoporosis in broilers by affecting calcium absorption, causing immune deficiencies, impairing HPA function, and inducing oxidative stress in bone cells [29]. Research conducted on White Shell Roman layers using an animal model of osteoporosis has shown lower indicators of bone biomechanical strength and mineral density, along with elevated levels of 5-HT in the high-fat group compared to control subjects [7]. It has been indicated that 5-HT serves as a crucial neurotransmitter linking the brain–gut axis. There are two types of responses involved in the regulation of bone health: the neurotransmitter form of brain-derived serotonin and the hormone form of gut-derived serotonin, which have opposing effects on osteogenesis [30]. Interestingly, heat shock factor 1 (HSF-1) in the nervous system may delay aging by regulating the brain–gut–axis-mediated bone morphogenetic protein (BMP) signaling pathway [31]. BMP is part of the transforming growth factor beta (TGF-β) superfamily, which plays a significant role in the development and repair of cartilage and bone [32]. Given this, it is reasonable to propose that HSF-1 regulates the aging process by influencing bone or cartilage through the brain–gut axis.

On the other hand, the gut–bone axis actively regulates bone resorption and formation through intestinal metabolites, short-chain fatty acids, vitamins, bile acids, and their derivatives. Therefore, it is reasonable to conclude that incorporating probiotics into poultry diets can effectively prevent skeletal diseases [33]. For instance, research indicates that administering *Bacillus globulus* directly to turkeys enhances bone mineralization and lowers serum peptide YY concentrations (a gut hormone that inhibits appetite). Additionally, *Bacillus globulus* aids in reducing inflammation, maintaining gut integrity, and promoting beneficial microbial interactions, all of which may contribute positively through the gastrointestinal tract [34]. The gastrointestinal tract houses trillions of microorganisms, collectively known as the gut microbiome, which maintain a symbiotic relationship with their host. In broiler chickens, the dietary inclusion of *Clostridium butyricum*-based probiotics has been shown to alter the composition of cecal microbiota, influencing the secretion of gut–brain peptides and hormones that help regulate bone metabolism by increasing levels of 5-HT [35]. In laying hens, dietary supplementation bacillus with subtilis-based probiotics has been associated with increased expression of the femoral TNF receptor superfamily member 11b (osteoprotegerin (OPG)), reduced bone damage [36], and enhanced levels of beneficial bacteria in the cecal microbiota. Overall, probiotics have demonstrated promising therapeutic effects on poultry bone health [37].

In summary, the brain–gut–bone axis is a dynamic system regulated by numerous signaling pathways. NPY, neurotransmitter (5-HT), HSF-1, and the HPA axis directly influence osteoclast activity in osteoblasts, thereby impacting the balance between bone formation and resorption. 5-HT plays a dual role in bone formation and is significantly affected by gut microbiota. However, the widespread application of these findings in production and the in-depth investigation of underlying mechanisms remains limited. Due to its complex hormonal interactions, this knowledge could establish a foundation for clinical conversion points based on the therapeutic targets of neuropeptides and neurotransmitters.

## 3. Gut–Bone Axis: Bridging Bone Health Through Metabolites

### 3.1. Short-Chain Fatty Acids and Bone Health

The gut microbiota has been shown to influence bone metabolism by producing metabolites, including bile acids (BAs), tryptophan metabolites, and short-chain fatty acids (SCFAs). These metabolites enter the bloodstream, representing one way the gut microbiota regulates bone homeostasis in chickens [38]. The influence of gut microbiota and its associated metabolites on bone health is often referred to as the gut–bone axis. SCFAs are essential microbial metabolites produced through the bacterial fermentation of carbohydrates. They bind to various G protein-coupled receptors (GPRs) on intestinal endothelial cells, known as free fatty acid receptors (FFARs) [39]. This binding allows SCFAs to affect these cells and facilitates their transport throughout the body. Additionally, SCFAs are absorbed into the bloodstream via passive diffusion, enabling them to impact distant tissues, including bones, muscle, and the liver [40,41].

Research indicates that SCFAs can enter the bloodstream by binding to colonic epithelial transporter proteins, specifically SMCT1/Slc5a8 (sodium-driven) and MCT1/Slc16a1 (proton-driven). These transporters facilitate SCFA movement into the portal vein and hepatic circulation [42]. Furthermore, SCFAs play distinct roles in various tissues: they serve as substrates for energy production in intestinal epithelial cells, particularly butyrate; in colon cells, both butyrate and propionate enhance the absorption process; and in intestinal cells, propionate is involved in the gluconeogenic pathway as a sub-strate for intestinal gluconeogenesis [43,44]. Perhaps most notably, the 150 mM SCFA therapeutic supplement may be a powerful tool in balancing osteoclast activity and the inhibition of bone resorption [45].

**Figure 2:**
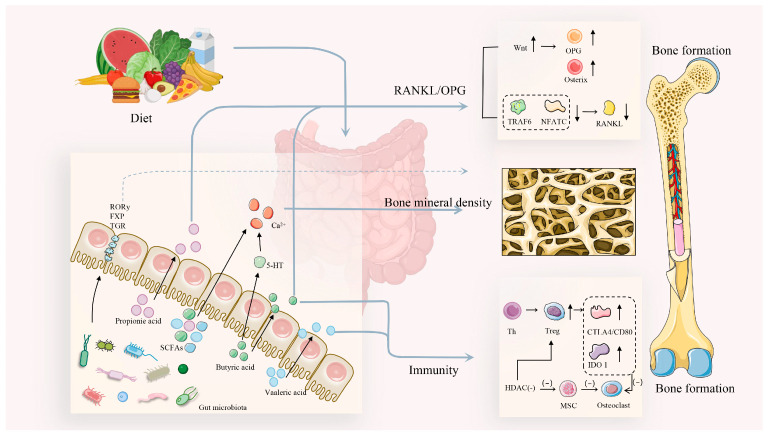
The gut–bone axis: connecting bone health through short-chain fatty acids (SCFAs). Short-chain fatty acids (SCFAs), including butyric, propionic, and valeric acids, play a significant role in bone metabolism. Butyric acid stimulates the production of 5-HT, which acts synergistically with calcium ions generated during SCFA metabolism to influence bone density. Butyric and valeric acids collectively modulate bone resorption in an immune context. The combined action of Th cells and HDAC increases Treg cell populations, subsequently elevating CTLA4/CD80 and IDO1 levels, which, in turn, reduce osteoclast activity. Additionally, reduced HDAC activity decreases the bone marrow mesenchymal stem cell (MSC) population, further negatively regulating osteoclasts. Butyric and propionic acids promote bone formation through the RANKL/OPG pathway, elevating OPG and osterix while reducing RANKL levels. Abbreviations: Treg, regulatory T cells; Th, helper T cells; HDAC, histone deacetylase; IDO1, indoleamine 2,3-dioxygenase 1; MSC, mesenchymal stem cells; OPG, osteoprotegerin; TRAF6, TNF receptor-associated factor 6; NFATC, nuclear factor of activated T cells.

In the first part, SCFAs act on osteoblasts via immune pathways or the Wnt pathway to promote bone formation (Figure 2). For example, SCFAs, such as acetate, can enhance calcium absorption, decrease bone resorption, and promote bone formation, depending on their concentration and their ratio to other microbial SCFAs, like propionate and butyrate [45]. Strategies to enhance the activity of probiotic strains present opportunities for nutritional solutions to improve or maintain bone health. Research has shown that treatment with prebiotics (such as galacto-oligosaccharides) and probiotics (like *Limosilactobacillus reuteri*) increases the production of SCFAs, including lactate and acetate, and that their preconditioned supernatants can elevate several osteogenic differentiation markers and stimulate bone mineralization. This suggests that probiotics influence bone health by regulating SCFA levels [46]. Furthermore, the activation of Wnt/β-catenin signaling following sodium butyrate treatment in terminally differentiated broilers resulted in elevated concentrations of osteogenic markers such as Runt-related transcription factor 2 (Runx2) and Osteopontin (OPN), positively regulating osteoblasts and terminal hypertrophic chondrocytes [47]. Additionally, supplementation with *Clostridium butyricum*, a butyrate-producing probiotic, was beneficial for tibia development in pullets and upregulated alkaline phosphatase and Runx2 levels. From an immunological perspective, *Clostridium butyricum* significantly increased IL-10 expression and enhanced the proportion of T regulatory (Treg) cells in the spleen and peripheral blood lymphocytes, which stimulated higher levels of bone formation in vitro [48]. These studies indicate that butyrate, either through dietary supplementation or produced through a probiotic, benefits bone health in poultry; however, Song et al. [48] did not assess whether *Clostridium butyricum* supplementation led to increased fecal butyrate levels.

The second part indicates that SCFAs inhibit osteoclast-mediated bone resorption and modulate calcium–phosphorus metabolism through inflammatory cytokines, ultimately favoring bone formation over resorption. A study on bone health in broiler ducks demonstrated that the dietary inclusion of resistant potato starch led to the production of SCFAs in the cecum, resulting in increased levels of propionic acid and butyric acid. Notably, there were significant reductions in markers of osteoclastic activity, such as tartrate-resistant acid phosphatase (TRAP) and CTx, as well as in the receptor activator of nuclear factor κB (RANKL)/OPG ratios. These findings suggest that SCFA production enhances tibial bone quality and strength by inhibiting osteoclast-mediated bone resorption driven by inflammatory cytokines [49]. Another study demonstrated that SCFAs mitigate bone loss by decreasing osteoclast bone resorption activity [50]. Broiler bone health is closely related to calcium (Ca) and phosphorus metabolism. Supplementation with zinc in broilers leads to increased lactate concentrations, which serve as an energy source for bacteria, facilitating the synthesis of SCFAs such as acetate, propionate, and butyrate. This indicates that SCFAs can directly influence bone health through their interaction with calcium [51]. It is clear that short-chain fatty acids have a broad range of pathways that impact bone health in the gut, both directly by enhancing bone mass and indirectly through their effects on the immune system, calcium absorption, and the activity of osteoblasts and osteoclasts, thus affecting bone metabolic activity [52].

### 3.2. Bile Acids and Bone Health

Bile acids, the primary component of bile, have recently attracted attention for their role in the pathology of bone diseases caused by gut microbiota dysbiosis. These amphiphilic cholesterol metabolites are synthesized by cholesterol 7α-hydroxylase (CYP7A1) in the classical pathway within hepatocytes or sterol 27-hydroxylase (CYP27A1) in the alternative pathway in extrahepatic tissues. Bile acids act as signaling molecules in the intestine by promoting the absorption of lipids and fat-soluble vitamins, regulating glucose and lipid homeostasis, and maintaining the balance of intestinal microbiota [53,54]. Bile acids can be classified into primary and secondary bile acids based on their source. Primary bile acids are produced in the liver from cholesterol breakdown, while secondary bile acids are formed through further metabolism by gut microbiota. Both types, along with their oxygenated derivatives (oxo-bile acids, hydroxylated bile acids, and carboxy bile acids), have been identified as signaling molecules interacting with bile acid-activated receptors [55,56]. Furthermore, bile acids exhibit hormonal properties mediated by specific receptors, most notably the farnesoid X receptor (FXR) and the G protein-coupled bile acid receptor 1 (GPBAR-1, also known as TGR5). Other receptors, such as the liver X receptor (LXR), pregnane X receptor (PXR), constitutive androstane receptor (CAR), vitamin D receptor (VDR), and sphingosine-1-phosphate receptor 2 (S1PR2), are also activated by bile acids in conjunction with various more selective endogenous ligands [57,58].

Bile acids are recognized as the most potent natural agonists for TGR5, which is widely expressed, particularly in the liver, intestine, brown adipose tissue, and immune cells. TGR5 has been implicated in the regulation of multiple metabolic functions, particularly energy and glucose homeostasis [58]. Research indicates that supplemental bile acids in animal diets can enhance nutrient digestibility and growth performance and even promote hepatic biotransformation and excretion of aflatoxin B1 [59,60]. Recent data also point to the involvement of bile acids in bone metabolism [61,62]. In postmenopausal women, it has been observed that serum total bile acid levels were significantly lower in the osteoporosis and osteopenia groups than in healthy controls (*p* = 0.002 and *p* = 0.018, respectively), and serum bile acid levels were positively correlated with the BMD of the lumbar spine, the femoral neck, and the total hip [63,64]. It is unclear whether bile acids actively regulate BMD, perhaps only in relation to metabolic changes in osteoporosis. In one study, the treatment of macrophages with the differentiated bile acid receptor agonist SH-479 (IC≈ 0.1 μM) on days 1, 3, and 5 revealed a potent inhibition of RANKL-induced osteoclast differentiation on day 1; 500 nM SH-479 was also found to enhance osteoblast differentiation and mineralization [65]. Therefore, the role of bile acids as adjuvants in regulating bone metabolism should not be overlooked.

Osteoblasts, the primary builders of bone tissue, are chiefly responsible for the synthesis and secretion of the bone matrix. Bile acids have been shown to significantly regulate the proliferation and differentiation of osteoblasts by binding to specific receptors. Some studies have found that the farnesoid X receptor (FXR) and the G protein-coupled receptor 5 (TGR5) are widely expressed in various types of bone cells [66,67]. This may imply that FXR activation has a beneficial effect on bone formation. Furthermore, the activation of FXR and TGR5 reduces osteoclastogenesis, inhibits essential osteoclast genes, such as c-Fos and nuclear factor of activated T cells cytoplasmic 1 (NFATc1), and promotes the expression of osteoblast-associated signaling pathways upon binding to bile acids [66,67]. The gut microbiota has been identified as a crucial contributor to host metabolism. Increasing evidence indicates that the gut microbiota, along with their metabolites, such as bile acids and SCFAs, play an increasingly significant role in the regulation of bone homeostasis via a direct gut–bone signaling axis [61]. For instance, antibiotic treatment in Specific Pathogen-Free (SPF) mice has shown an increase in serum-conjugated bile acids that act as FXR antagonists, suppressing osteoblast function, decreasing bone mass, and impairing bone microarchitecture and fracture resistance. This suggests that they may serve as novel mediators of gut–bone signaling, modulating the dynamic balance of the bone matrix. Notably, TGR5 agonists can enhance alkaline phosphatase (ALP) activity, promote matrix mineralization, and increase the expression of osteogenic marker genes, including osteocalcin, collagen type I (Col-I), ALP, osterix (an essential transcription factor for osteoblast differentiation and bone mineralization), and Runx2.

Bone tissue absorbs bile acids from the serum and releases them into the bone microenvironment during uptake by passive diffusion rather than active transport. One study investigated the impact of bile acids on bone resorption and osteoclast activity [61]. Another study reported that bile acids function as physiological ligands, targeting FXR on osteoclasts and effectively activating the transcription of FXR target genes [68]. The FXR/fibroblast growth factor 15 axis acts in vivo as an intestinal/hepatic endocrine axis for bile acid homeostasis. Impaired FXR signaling has been linked to reduced fracture resistance and bone mass after minocycline treatment, further elucidating the role of bile acids in introducing the intestinal–hepatic signaling pathway indirectly in the skeleton [69]. These studies suggest that bile acids, their metabolites, and bile acid receptors within the gut microbiota may be central mechanisms for promoting bone health. In recent years, bile acid supplementation has improved nutrient digestibility and growth performance, decreased abdominal fat deposition, and enhanced hepatic biotransformation and excretion of aflatoxin B1 [59,60]. However, whether bile acid supplementation can improve bone health in poultry, along with the underlying molecular mechanisms, remains largely unknown (Table 1).

### 3.3. Tryptophan Metabolites and Bone Health

Tryptophan is considered one of the essential amino acids required for optimal growth performance and feed utilization in poultry, along with lysine and methionine [74]. It has been demonstrated that tryptophan enhances growth performance, reduces stress, regulates insulin, and improves meat quality in poultry, as it serves as a precursor for niacin, melatonin, and serotonin [75]. The three primary metabolic pathways of tryptophan are the indole pathway, the 5-HT pathway, and the kynurenine pathway. Additionally, tryptophan metabolism plays a critical role in the gut microbiota, where microorganisms convert tryptophan into indole and its derivatives. These compounds help maintain a dynamic equilibrium in the gut microbiota through the regulation of pro- and anti-inflammatory cytokines [76]. High levels of tryptophan supplementation also stimulate melatonin synthesis, enhancing the innate immune response and antioxidant status in broilers [77]. Notably, dietary tryptophan significantly affects both drip loss and the shear force of breast muscles. Moreover, it positively regulates the balance between pathogenic and non-pathogenic gut bacteria. This indicates that in transported broiler chickens, a tryptophan level of 0.42% or higher can be beneficial before and after stress, as it helps to reduce serum corticosterone and heat shock protein 70 levels while elevating the 5-HT level, thereby mitigating the adverse effects of stress [78]. Additionally, in white geese aged one to twenty-eight days, tryptophan has been shown to facilitate the development of the proximal intestine and the deposition of breast meat protein [79]. These studies underscore the essential role of tryptophan in poultry development, immunity, and stress response.

Tryptophan metabolism is involved in a wide range of biological processes, with the primary pathway in the liver being degradation via the kynurenine pathway. Approximately 95% of dietary tryptophan is metabolized through this pathway, with approximately 90% occurring in the liver. This process is catalyzed by the enzymes indoleamine-2,3-dioxygenase (IDO, including both IDO1 and IDO2), which mediate the initial and rate-limiting steps of the pathway. IDO1-mediated tryptophan metabolism produces kynurenine, which plays a crucial role in promoting Runx2 ubiquitination. A deficiency in kynurenine negatively affects IDO1 ablation, leading to increased Runx2-mediated osteogenic reprogramming and calcification of vascular smooth muscle cells [80]. In recent years, significant interest has been observed in investigating the effects of high-fat diets on osteoporosis, as dietary fat contributes to pathological alterations in bone tissue. Tryptophan signaling is activated by a high-fat diet, which further increases the 5-HT/ERK/CREB pathway, accelerating bone resorption associated with osteoclast differentiation [7]. Generally, the application of tryptophan in poultry has focused on improving dietary utilization, reducing environmental stress, enhancing immune function, and increasing production performance. In contrast, research concerning poultry bone health requires further exploration [81,82,83]. Conversely, the effects of tryptophan on bone health have been more extensively studied in mice and humans [84]. For instance, after exposure to high doses of ionizing radiation, the tryptophan metabolite indole-3-carboxaldehyde has been shown to prolong healthy lifespan, accelerate peripheral blood cell repair, and help mitigate ionizing radiation-induced myelosuppression in mice [73]. Additionally, *Bacillus shortis*-mediated indole-3-lactic acid prevents colitis and tumorigenesis by inhibiting macrophage differentiation [85]. Based on the studies mentioned, exploring the interaction between tryptophan and its metabolites in relation to poultry bone health may represent a promising direction for future research (See Table 1).

### 3.4. 1,25-Dihydroxyvitamin D3 and Bone Health

Vitamin D signaling plays a crucial role in maintaining intestinal homeostasis and is traditionally linked to bone health by enhancing the absorption of dietary calcium and mobilizing stored calcium from the bones [86]. Calcium-sensing receptors (CaSRs) found in the gastrointestinal tract are sensitive to calcium concentrations, regulating thyroid hormone secretion from the thyroid gland, which, in turn, governs vitamin D synthesis and influences calcium handling in the intestines, bones, and kidneys [87]. Calcium absorption occurs predominantly in the small intestine and is facilitated in the intestinal mucosal epithelium via transcellular and paracellular pathways, regulated by 1,25-Dihydroxyvitamin D3 (1,25(OH)_2_D_3_) and calcitriol. In the presence of phosphate, absorbed calcium ions reach the skeleton through the bloodstream, following intestinal absorption and renal reabsorption, and play a role in poultry bone metabolism.

**Figure 3:**
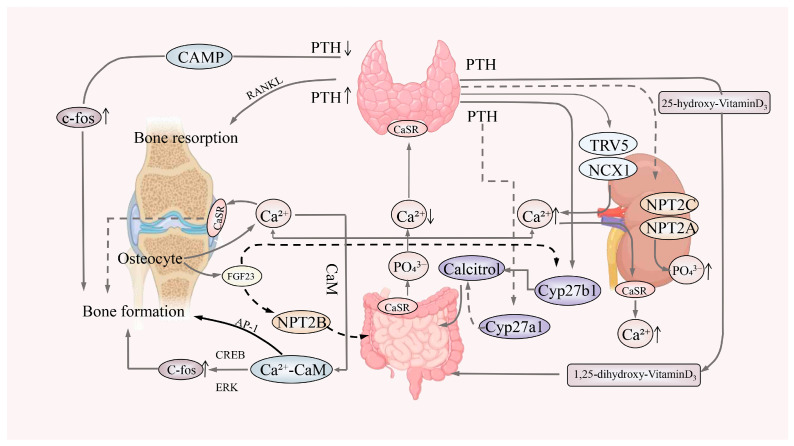
Gut–bone axis: connecting bone health through 1,25-Dihydroxyvitamin D3. 1,25-Dihydroxyvitamin D3 and parathyroid hormone (PTH) work synergistically to maintain calcium homeostasis. The thyroid produces PTH, stimulating the release of phosphate transporters NPT2C and NPT2A in the kidney, promoting phosphate excretion (PO_4_^3−^). PTH also enhances the release of calcium ions (Ca^2+^) via TRV5 and NCX1 in the kidney, facilitating urinary calcium excretion. Elevated Ca^2+^ levels bind to the calcium-sensing receptor (CaSR) in the kidney, promoting bone resorption. Concurrently, the release of Ca^2+^ from the bone surface can stimulate bone formation. PTH promotes bone resorption through the RANKL pathway while enhancing bone formation via increased c-fos expression. Intestinal-derived Ca^2+^ forms a complex with calmodulin (CaM), which augments the expression of c-fos and AP-1, thereby promoting bone formation. Abbreviations: PTH, parathyroid hormone; FGF-23, fibroblast growth factor 23; Cyp27b1, cytochrome P450 27B1; Cyp27a1, cytochrome P450 27A1; c-fos, cellular proto-oncogene fos.

1,25(OH)_2_D_3_ is synthesized from inactive vitamin D3 in the skin through the action of hydroxylases in the liver and kidneys (Figure 3). It stimulates osteoblast activity, promoting the deposition and mineralization of bone salts while simultaneously increasing osteoclast activity, leading to bone resorption in chickens [88]. However, gut bacterial dysbiosis, which can lead to enteritis, may result in inadequate calcium absorption and reduced circulating levels of vitamins D and K, potentially leading to decreased bone mass [89]. The gut microbiota also regulates bone metabolism in relation to dietary intake. Carbohydrates consumed in the diet are fermented by gut microbiota to produce SCFAs, which enhance bone metabolism by increasing calcium absorption and improving the mineral solubility of calcium and phosphate [90]. Research has shown that sclerostin expression promotes intestinal and renal reabsorption of calcium, further increasing bone density; the presence of sclerostin can directly or indirectly influence bone quality through the 1,25(OH)_2_D_3_–intestinal–kidney axis [91].

Furthermore, a study confirmed that exposure to aflatoxin (AFB1) at levels exceeding 230 ppb drastically downregulated the mRNA expression of the vitamin D receptor and calcium–phosphorus transport proteins in the jejunum, adversely affecting broiler bone health [92]. In addition, deoxynivalenol contamination in piglet diets disrupts calcium and phosphorus metabolism, resulting in the downregulation of genes involved in intestinal and renal calcium and phosphorus absorption, alongside increased bone mineralization [93]. These studies underscore that changes in the gut microbiota are closely associated with alterations in calcium absorption and bone-related indicators. Nonetheless, there is currently a lack of research investigating the potential for supplementing with 1,25(OH)_2_D_3_ or pharmacological interventions to enhance intestinal and renal absorption of 1,25(OH)_2_D_3_ in order to promote osteogenesis in poultry suffering from leg disease.

### 3.5. Inflammatory Cytokines and Bone Health

Bone metabolism is regulated by hormonal and local factors within the bone microenvironment. Recent studies have also revealed that the immune system plays a significant role in maintaining bone homeostasis. Proinflammatory cytokines, such as tumor necrosis factor-α (TNF-α) and interleukin-6 (IL-6), are crucial pathogenic factors associated with immune-mediated bone diseases. The gut microbiota interacts with and shares a wealth of molecular and regulatory mechanisms between the bone and immune systems, emerging as a key player in regulating bone turnover. It achieves this by modulating the immune system, controlling levels of inflammatory cytokines, and influencing calcium absorption and vitamin D levels [94]. Furthermore, the balance between the GM and helper T lymphocyte 17 (Th17) and T regulatory (Treg) cells is closely related to bone metabolism [95]. Th17 cells expand and migrate to the joints driven by intestinal dysbiosis, leading to bone loss through effects on osteoblasts and the production of RANKL. In contrast, stimulation by TNF-α, interleukin-17 (IL-17), and macrophage colony-stimulating factor (M-CSF) are primary drivers of osteoclastogenesis [96]. Conversely, Treg cells primarily influence bone resorption by preventing the differentiation of monocytes into osteoclasts [97].

Notably, alterations in intestinal integrity have been associated with decreased markers of bone resorption and increased levels of inflammatory cytokines such as IL-6 and TGF-β in poultry. These changes may be linked to inflammation-mediated variations in the mechanical properties of the tibia [98]. Osteoclast counts have also shown correlations with immune cell populations, specifically CD4^+^ T cells, as well as with intrabone levels of IL-6, RANKL, and TNF-α [99]. Maintaining the homeostasis of the gut microbiota is, therefore, critical for bone health in poultry. In an experimental study, laying hens treated with calcium butyrate exhibited reduced serum levels of IL-1 and TNF-α alongside increased serum levels of TNF-β and the immune mediator IgA [100]. This treatment enhanced their intestinal and systemic immune functions. Although there were no significant changes in tibia mass or mineral content, improvements were observed in tibia microstructure and mechanical properties [101]. From these observations, we can speculate that imbalances in the gut microbiota lead to altered levels of immune factors, which subsequently impact bone remodeling.

**Figure 4:**
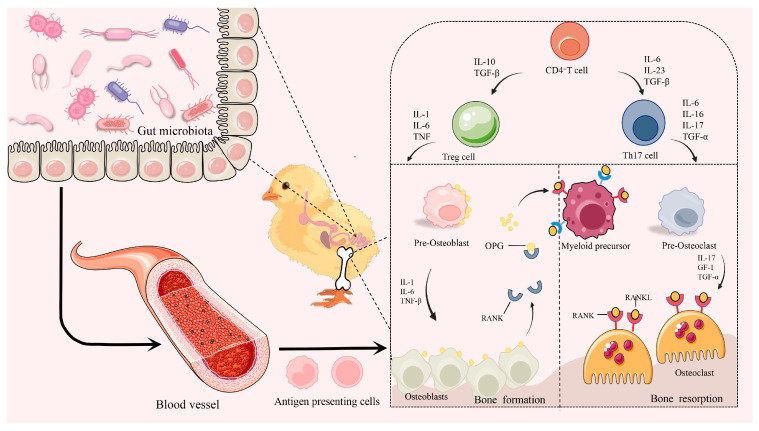
Gut–bone axis: connecting bone health through the bone immune system. Alterations in gut microbiota activate antigen-presenting cells (APCs), further stimulating CD4^+^ T cells. Regulatory T (Treg) cells enhance bone formation by secreting interleukins (IL-1 and IL-6) and tumor necrosis factor (TNF), influencing bone precursor cells. In contrast, Th17 cells promote osteoclast differentiation, leading to increased bone resorption. Abbreviations: APC, antigen-presenting cell; IL, interleukin; Pre-OB, pre-osteoblast; OPG, osteoprotegerin; OB, osteoblast; Pre-OC, progenitor cell of osteoclast; RANKL, receptor activator of nuclear Kappa-B ligand; RANK, receptor activator of nuclear Kappa-B; OC, osteoclast.

The integrity of the intestinal barrier, particularly in the context of inflammation, can also influence bone development in poultry [98]. This phenomenon may be due to systemic inflammation caused by bacterial translocation and the invasion of toxins resulting from the leakage of intestinal fluids following damage to the intestinal barrier. This process is associated with both inflammatory and immune-mediated osteoporosis and arthritis and may contribute to lower bone quality in poultry [102]. These findings underscore the significant influence of gut microbiota on poultry bone health through osteoimmunological mechanisms (Figure 4).

## 4. Gut–Organ Axis: Driving the Distal Organs for Bone Health

### 4.1. Gut–Kidney Axis and Bone Health

Traditional Chinese medicine believes that “the kidneys govern the bones”. During the Ming and Qing dynasties, the “Surgical Prescription Medicine Tongchange Prescription” articulated the theory that “the kidneys are fundamentally linked to bone vitality”. This implies that the growth, development, and functionality of bones are closely associated with the normal functioning of the kidneys. Calcium and phosphorus metabolism balance is essential for maintaining bone homeostasis, with the kidneys playing a critical role in their reabsorption and excretion. After glomerular filtration, over 80% of calcium and phosphorus are reabsorbed in various segments of the renal tubules [103]. Moreover, it has been demonstrated that the processes involved in systemic calcium–phosphorus homeostasis are influenced by ingested calcium (Ca^2+^) and phosphate, mediated through the co-regulation of intestinal, renal, and bone mechanisms—especially prominent in cases of renal insufficiency and bone abnormalities [104]. Achieving a balance between bone resorption and formation in poultry thus requires a coordinated approach to calcium and phosphorus metabolism (Figure 3).

Parathyroid hormone [105], calcitriol (a protein that enhances intestinal calcium and phosphorus absorption, regulates renal calcium and phosphorus reabsorption and excretion, and promotes bone growth and remodeling) [106], and fibroblast growth factor 23 (FGF-23) [107] are the primary hormones regulating phosphate turnover in bone [108]. Research has shown that treatment with calcitriol, even after 48 weeks, can lead to decreased markers of bone formation—specifically, the propeptide of type I procollagen (PINP)—and markers of bone resorption (CTX) in patients with chronic kidney disease. This indicates that calcitriol influences bone remodeling in kidney patients experiencing imbalances in calcium and phosphorus metabolism [109]. Calcium homeostasis is regulated by 1,25 (OH)_2_D_3_, which predominantly acts at the gut level, as well as thyroid hormone, which exerts rapid and direct effects at both the kidney and bone levels [110]. Another study demonstrated that 1,25 (OH)_2_D_3_ and parathyroid hormones (PTHs) affect the expression of FGF-23 mRNA in chicken osteoblasts. FGF-23, an inhibitor of bone remodeling, was shown to reduce the activity of alkaline phosphatase (ALP) through the FGF-23-ERK pathway, further inhibiting the osteogenesis of BMSCs [111]. These studies collectively indicate that the gut microbiome exerts a significant impact on poultry bone health through the kidney system.

The kidneys play a crucial role in urine production and detoxification and maintaining electrolyte balance. Additionally, they indirectly regulate calcium and phosphorus metabolism to support bone structure and function through the regulation of BMP-7 [112], FGF-23 (a bone-derived endocrine factor), OPG [113], osteocalcin (a protein hormone produced by bones), and other hormones. Unlike rats and mice, chicken kidneys do not express BMP-7; however, it is readily detected in growth plates and cartilage [114]. FGF-23, a negative regulator of bone remodeling, is involved in phosphate reabsorption and vitamin D hormone production in the kidneys. It also inhibits bone formation and osteoblast mineralization of BMSCs via the FGFR3-ERK signaling pathway [111]. On day 52, the bone formation markers ALP, dentin matrix protein1 (DMP1), and sclerostin showed higher abundance in the groups receiving combinations of AT5 or BT4 (two different dietary vitamin D combinations). Some forms of vitamin D regulate the absorption and reabsorption of Ca and P in the intestine and kidneys, suggesting that these vitamin D combinations can directly regulate bone remodeling. Bone mineral content may also be influenced by the balance of Ca and P homeostasis [115]. Moreover, the kidneys directly impact bone tissue, promoting bone growth and development. Dietary studies with varying doses of Ca/P ratios indicate that transient receptor potential cation channel subfamily C member 3 (TRPC3) and cytochrome P450 family 24 subfamily A member 1 (CYP24A1) genes, which are involved in calcium and vitamin D homeostasis, are differentially expressed in the kidneys. Notably, the humerus length in the high P ratio group is slightly shorter than in the medium P ratio group, suggesting that the Ca/P ratio may significantly influence bone mineral reabsorption and formation [116]. ALP serves as a marker for bone and kidney health, and the combination of vitamin K3 and *Bacillus subtilis* alters the expression of ALP and related osteogenic genes, thereby enhancing tibia strength [117]. However, the specific mechanisms underlying the transport of calcium and phosphate ions among the intestine, kidneys, and bones remain unclear. Most existing studies have been conducted in mice, limiting our ability to generalize these findings. Therefore, further research involving a broader range of subjects is essential to determine whether a direct correlation exists within the intestinal–kidney–bone axis.

### 4.2. Gut–Liver Axis and Bone Health

The liver serves as the body’s metabolic center, interacting with various organs to maintain homeostasis. It is a vital organ involved in nutrient metabolism, immunity, intestinal homeostasis, cytokine production, and hormone regulation [118]. Inflammatory cytokines triggered by impaired liver function, hormones, and imbalances in gut microbiota cause the downregulation of certain factors in the liver, which may ultimately lead to an imbalance in the activity of osteoblasts and osteoclasts, which can lead to bone loss. Osteoporosis, osteopenia, and fractures are common bone complications associated with chronic liver disease [119]. However, the mechanisms underlying chronic liver disease are complex and multifactorial, with many details still unclear. This component can be distinguished based on factors that play different roles in the liver–bone axis.

First, we evaluated inflammatory cytokines that are upregulated in the presence of liver damage and affect bone metabolism, specifically IL-6 and TGF-α [120]. Previous studies have shown that the expression of IL-6 and TNF-α increases during liver injury in broilers, subsequently impacting bone metabolic disease [121]. Additionally, clinical studies have demonstrated that supplementation with probiotics or Chinese herbal extracts reduces IL-6 and TNF-α levels, potentially due to their ability to enhance antioxidant capacity and improve gut microbiota [122,123]. Therefore, it can be inferred that probiotics and Chinese herbal extracts positively influence liver and bone health. Encouragingly, numerous studies have demonstrated that herbs and herbal extracts—such as *Morinda officinalis* polysaccharides and the total flavonoids of *Rhizoma Drynariae*—can positively improve the gut microbiota of broilers by increasing the number of beneficial bacteria to restore tibial growth plates and increase tibial weights [9,124,125,126]. In vivo research has shown that dietary cannabis seeds can increase liver tocopherol levels and positively affect rooster tibia health [127]. Although this study did not detect the expression of IL-6 and TNF-α in the liver, its findings are consistent with our inference. Both IL-6 and TNF-α induce immune stress, stimulating osteoclast production [128], a phenomenon also observed in obesity-induced osteoporosis [129]. Based on the above in vivo and in vitro findings, we can hypothesize that probiotics or herbal extracts reduce IL-6 and TNF-α levels by improving the gut microbiota to slow down the corresponding bone damage diseases.

Secondly, chronic liver disease is associated with metabolic bone disease, characterized by changes in mineral density and manifesting as osteomalacia or osteoporosis. Deficiency of the metabolic regulator vitamin D is closely related to osteoporosis [130], and FGF-21, as a negative regulator of bone mass, also plays a role in metabolic processes [131]. Vitamin D deficiency is commonly associated with liver disease [132]. Patients with hepatitis B cirrhosis have exhibited reduced levels of 25-hydroxyvitamin D3, impaired liver function, and elevated levels of IL-6, TNF-α, and IL-1, which may be linked to decreased bone mineral density (BMD) [130]. The expression of FGF-21 and PGC-1α is upregulated in mice, suggestive of non-alcoholic fatty liver disease [133], but FGF-21 does not appear to be similarly expressed in poultry [134]. Given that current research on poultry liver diseases is still in the discovery phase [135,136], and the relationships between vitamin D, FGF-21, and bone health in liver disease have not shown the same trends as in mice or humans, further exploration of the molecular mechanisms of the liver–bone axis is necessary.

Finally, attention has been given to BMP-9 [137] and insulin-like growth factor (IGF-1) [138], both of which are closely related to bone formation, as well as sclerostin-related factors that promote bone resorption. BMP-9, produced by the liver, has been shown to be expressed in both the intestine and the liver [139]. Translocating the expression of a BMP-9 mutant in a chicken micromass culture has been found to increase chondrogenic activity in a chick model [140]. In vitro experiments indicate that IGF-1 secreted by hepatocytes plays a significant role in the biosynthesis of chondrocytes in the growth of chickens by participating in the transport of inorganic phosphate [141]. Genome-wide association studies suggest that the sclerostin gene is a strong candidate for investigating osteoporosis in chickens [142]. However, current studies indicate that the sclerostin gene is expressed in the liver rather than the intestine, and there are no clinical studies addressing the relevant indicators of inhibiting osteogenesis [143,144]. In summary, poultry clinical studies have not thoroughly explored the complex interactions between gut microbes, the liver, and bone. Specifically, due to an incomplete understanding of molecular pathways, the roles of FGF-21, BMP-9, and bone metabolism in poultry remain unclear and limited (Table 2).

### 4.3. Gut–Pancreatic Axis and Bone Health

The pancreas functions as both an exocrine and endocrine gland. The exocrine function involves secreting pancreatic juices rich in digestive enzymes, while the endocrine component involves insulin and glucagon secretion by islets distributed throughout the pancreatic tissue. Pancreatic juices break down nutrients such as starch, fat, and protein, aiding digestion and absorption. Endocrine insulin plays a role in regulating blood sugar levels and maintaining glucose homeostasis [152]. The biological response between insulin and bone relies on binding insulin receptors (IRs), which are abundant in osteoblasts [153]. Specifically, IRs are expressed in both osteoblasts and osteoclasts [154], but insulin receptor substrate (IRS)-1 promotes bone synthesis during bone remodeling. In contrast, the expression of IRS-2 in osteoclasts also influences bone formation [155]. In vitro experiments have demonstrated that insulin signaling in osteoblasts promotes bone resorption through osteocalcin decarboxylation [156]. Elevated insulin levels with the concomitant upregulation of IRs-1 were observed in an SDM (Steroid diabetes mellitus) rat model [157]; meanwhile, insulin levels were regulated by SCFAs and bile acids (gut microbiota metabolites) [158]. Changes in bone turnover markers have been observed following the inhibition of intestinal microbiota function [159]. This evidence supports the notion that gut microbiota may mediate bone health homeostasis by modulating insulin levels and binding IRs in osteoblasts. After a meal, beta cells in the pancreas secrete insulin, while intestinal endocrine cells release incretin hormones, such as glucagon-like peptide-1 (GLP-1) and gastric inhibitory polypeptide (GIP), which act on osteoblasts via the adenylate cyclase-cAMP pathway [160]. More importantly, research has shown that gut microbiota can disrupt blood glucose homeostasis, leading to reduced vascularization in tibial bones in broilers, resulting in tibial injury [151]. This finding suggests that the molecular mechanisms underlying the gut–pancreatic–bone axis in poultry are akin to those observed in mammals, offering a new perspective for future treatments of bone diseases in poultry (Table 2).

## 5. Conclusions

With increasing demand for domestic and international poultry, bone health issues have become a significant concern, jeopardizing growth rates and meat quality. The current research primarily focuses on the effects of the gut–brain axis on bone health in mice and humans; however, there is a pressing need for further understanding of the entero-osteotropic axis and its molecular mechanisms in poultry. The role of gut microbiota in poultry bone health is critical and cannot be overstated. The gut microbiota is regulated by the brain via the central nervous system and peripheral nerves. This regulation occurs not only directly through bidirectional communication between the brain and bones via neurotransmitters, such as serotonin (5-HT), norepinephrine, and HSF-1, but also indirectly by influencing gut microbiota metabolites, along with hormones and other factors that affect bone health.

**Figure 5:**
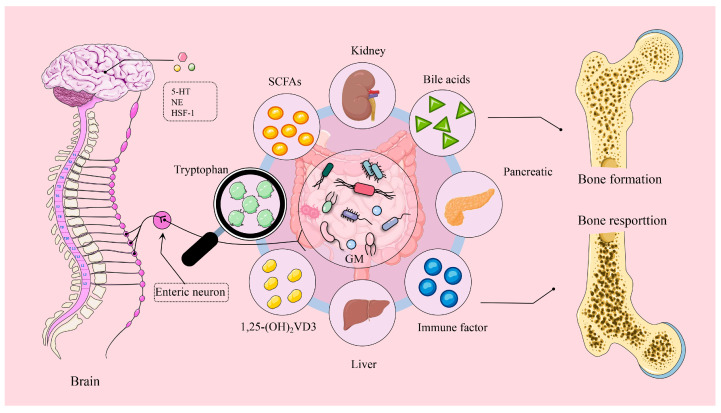
Brain–gut–bone axis: transmission of sensory signals that drive gut microbiota. Cerebral neurons regulate enteric neurons through the actions of 5-hydroxytryptamine (5-HT), norepinephrine (NE), and heat shock transcription factor 1 (HSF-1). In turn, enteric neurons affect bone health by regulating intestinal microbiota and their metabolites, influencing the liver, kidney, and pancreas.

As a central hub for regulating the balance of bone metabolism, the gut microbiota plays a crucial role in maintaining homeostasis and ensuring proper functioning between the brain and various organ systems (Figure 5). This article briefly reviews the intricate crosstalk mechanisms between the brain, gut microbiota and their metabolites, and bones, emphasizing the implications for bone health mechanisms and potential therapeutics in poultry. It is generally accepted that the primary causes of bone diseases in poultry are related to factors such as nutrition, genetics, dietary structure, and disease. Furthermore, many factors—including probiotics, herbs, dietary composition, and external stimuli—can directly alter the structure of gut microbiota. This presents a potential target for exploring the clinical application of combined pharmacological interventions or therapeutic approaches aimed at restoring gut microbiota homeostasis to improve poultry bone health [161].

However, systematically investigating these complex inter-relationships remains a significant frontier in biomedical research. Due to the limitations of current experimental techniques and methodologies, many factors and mechanisms involved in the gut–bone axis interaction still require further investigation. Additionally, the mechanisms underlying changes in bone mass in models of fatty liver disease need to be studied more comprehensively. To address these knowledge gaps in poultry bone health, we propose the utilization of new technological tools, such as nanomaterials and bone microarrays [162]. These advancements aim to pave the way for innovative therapeutic strategies and enhance our understanding of the biophysiology of poultry bone diseases.

## Figures and Tables

**Table 1:** Interaction of bile acids and tryptophan metabolites with bone metabolism.

Metabolite	Receptor	Target Cell	Effect	Function	References
Minocycline	FXR	Osteoblast	Inhibition	Inhibited osteoblast function, reduced bone mass, impaired bone microstructure and fracture resistance	[69]
Geniposidic acid	FXR	Osteoblast	Promotion	FXR is activated to promote osteoblast differentiation and stimulate bone formation	[70]
Folic acid	TGR5	Osteoblast	Promotion	Increased expression of LCA and TGR5, increased phosphorylation of AMPK, decreased phosphorylation of NF-κB and ERK, and reduced bone loss	[71]
Betulinic acid	TGR5 FXR	Osteoclast	Inhibition	Osteoclast differentiation is regulated by AMPK signaling pathway	[65]
Bile acid	TGR5	Osteoblast	Promotion	Promotes the expression of Runx2 and increases the expression of ALP, osteocalcin, osterix, and other osteogenic genes	[67]
Indole acetic acid (IAA) and indole-3-propionic acid (IPA)	Aryl hydrocarbon receptor	M2 macrophage	Promotion	Repair intestinal barrier function, promote osteoblast generation, and inhibit osteoclast generation	[72]
Tryptophan	5-HT	Osteoclast	Promotion	Activates 5-HT/ERK/CREB for bone resorption	[7]
Indole-3-carboxaldehyde	\	Peripheral blood cell	Promotion	Helps mitigate ionizing radiation-induced myelosuppression	[73]

**Table 2:** Gut–organ–bone axis factors that interact with bone metabolism.

Microbial Signal	Source	Disease/Model	Function	References
Kidney–Bone Axis				
Calcitriol	Kidney	Osteoarthritis	Inhibits the breakdown of extracellular matrix in osteoarthritis, early bone remodeling, and cartilage degeneration	[106]
Fibroblast growth factor 23 (FGF-23)	Osteoblast	Chronic kidney disease	Increased levels of FGF-23 and PTH and increased bone resorption	[107,145]
Bone morphogenetic protein (BMP)-7	Mesenchymal stem cells	Tibial damage	The number of tibial growth plate chondrocytes decreased, and osteoclast-related genes and enzymes increased. Chondrocyte differentiation was delayed, and BMP-6, BMP-7, SOX9, and Runx2 were downregulated	[112]
Osteopraxin (OPG)	Osteoblast	Tibial dyschondroplasia (TD)	Alkaline phosphatase (ALP) and osteocalcin levels were reduced	[113]
Parathyroid hormones (PTHs)	Parathyroid cells	Bone phosphorus retention and bone development	The level of FGF-23 was positively correlated with the level of PTH, and the ALP and bone gla protein (BGP) of tibia were increased	[105]
1,25-(OH)2-Vitamin D3	Kidney	In vitro study	Osteoblast differentiation is regulated by inhibiting Runx2 activity	[146]
Liver–Bone Axis				
BMP-9	Liver	Cranial defect model	Improves bone regeneration potential and enhances bone mechanical properties	[137]
Insulin-like growth factor 1 (IGF-1)	Liver	In vitro study	Upregulation of IGF-1 inhibits osteoblast apoptosis	[138]
Lecithin–cholesterol acyltransferase (LCAT)	Liver	Osteolysis	Inhibition of lecithin–cholesterol acyltransferase (IL-6) expression further inhibited the release of inflammatory cytokines from bone marrow macrophages, thereby inhibiting osteoclast formation and bone resorption	[147]
Tumor necrosis factor (TNF-α)	Mononuclear macrophage	Erosive arthritis	After treatment with TNF-α and RANKL, Bip blocked monocytes, and osteoclast precursor NF-κB signaling inhibits osteoclast generation	[120]
Recombinant sclerostin	Bone	Mouse embryonic fibroblast cell	Sclerostin gene silencing enhances the expression of osteogenic markers and promotes bone formation	[148]
LCAT	Liver	Hepatic osteodystrophy	Downregulation of LCAT expression exacerbates bone loss	[149]
Pancreatic–Bone Axis				
Insulin receptors (IRs)	Osteoblasts	Mouse calvarial osteoblasts	IGF-1 and IGFBP-2 coordinate to stimulate IRs-1 phosphorylation, osteoblasts, and AKT differentiation	[150]
Insulin	Pancreatic	TD	The level of glucose and pancreatic function were restored, and glycometabolism inhibited the expression of PI3K/AKT/VEGF pathway, which aggravated the lesions of TD	[151]

## Data Availability

The original contributions presented in this study are included in the article. Further inquiries can be directed to the corresponding authors.
